# CO_2_ and carbonate as substrate for the activation of the microbial community in 180 m deep bedrock fracture fluid of Outokumpu Deep Drill Hole, Finland

**DOI:** 10.3934/microbiol.2017.4.846

**Published:** 2017-10-23

**Authors:** Malin Bomberg, Mari Raulio, Sirpa Jylhä, Carsten W. Mueller, Carmen Höschen, Pauliina Rajala, Lotta Purkamo, Riikka Kietäväinen, Lasse Ahonen, Merja Itävaara

**Affiliations:** 1 VTT Technical Research Centre of Finland, P.O. Box 1000, FIN-02044 VTT, Finland; 2 Tikkurila Oyj, P.O. Box 53, Kuninkaalantie 1, FI-01301 Vantaa, Finland; 3 Lehrstuhl für Bodenkunde, Department Ecology and Ecosystem Management, Center of Life and Food Sciences Weihenstephan, Technische Universität München, D-85350, Freising-Weihenstephan, Germany; 4 Geological Survey of Finland (GTK), P.O. Box 96, 02151 Espoo, Finland

**Keywords:** groundwater, crystalline bedrock, NanoSIMS, FACS, methane, sulfate, *Pseudomonas*, autotroph, Outokumpu

## Abstract

Microbial communities in deep subsurface environments comprise a large portion of Earth's biomass, but the metabolic activities in these habitats are largely unknown. Here the effect of CO_2_ and carbonate on the microbial community of an isolated groundwater fracture zone at 180 m depth of the Outokumpu Deep Scientific Drill Hole (Finland) was tested. Outokumpu groundwater at 180 m depth contains approximately 0.45 L L^−1^ dissolved gas of which methane contributes 76%. CO_2_, on the other hand, is scarce. The number of microbial cells with intracellular activity in the groundwater was low when examined with redox staining. Fluorescence Assisted Cell Sorting (FACS) analyses indicated that only 1% of the microbial community stained active with the redox sensing dye in the untreated groundwater after 4 weeks of starvation. However, carbon substrate and sulfate addition increased the abundance of fluorescent cells up to 7%. CO_2_ and CO_2_ + sulfate activated the greatest number of microbes, especially increasing the abundance of *Pseudomonas* sp., which otherwise was present at only low abundance in Outokumpu. Over longer exposure time (2 months) up to 50% of the bacterial cells in the groundwater were shown to incorporate inorganic carbon from carbonate into biomass. Carbon recapture is an important feature in this ecosystem since it may decrease the rate of carbon loss in form of CO_2_ released from cellular processes.

## 1. Introduction

Precambrian shield areas, such as the Fennoscandian Shield, preserve the oldest geological records of our planet, but have also been affected by the continuous evolution of life, atmosphere, and climate. Processes taking place in the continental crust are not only of scientific interest, but microbial and geochemical processes in fractured crystalline rock play a key role also e.g., in assessing the safety of geological disposal of hazardous wastes. Microorganisms have a great impact on the elemental cycles in the upper crust and are responsible for the conversion of organic matter to be reused for the maintenance of the whole microbial community [1]. Carbon derived from photosynthesis only slowly diffuses into the terrestrial subsurface, if at all. Thus, alternative means of carbon assimilation, such as inorganic carbon fixation or methane oxidation may be important to support the microbial community.

The Outokumpu Deep Drill Hole provides access to study isolated groundwaters in the Paleoproterozoic part of the Fennoscandian Shield in eastern Finland [2]. The drill hole reaches a total depth of 2516 m and spans several fluid-filled fracture zones. The deep groundwater in Outokumpu is highly reducing and has low organic carbon content. Methane is the most abundant hydrocarbon and contributes up to 80% of the total volume of gas released from the groundwaters and is continuously discharging on the drill hole collar [3],[4]. The microbial communities in the Outokumpu deep subsurface have been reported to contain genes for different autotrophic carbon fixation pathways [5] and methane cycling [1],[6]. In addition, it has recently been shown that methane activates the microbial community residing in the fracture zone at 500 m in Outokumpu, but does not increase the number of active microbial cells in the fracture at 180 m [7]. Nevertheless, the transcription of bacterial 16S rRNA genes increased showing that especially *Pseudomonas* bacteria were affected by the added carbon substrates at 180 m depth, while at 500 m bacteria belonging to the order Rhodobacterales were activated [7]. Only one other study exists to date that show specific functional activity of the deep subsurface microbial community in Outokumpu [6]. Thus, more indication of actual microbial metabolic activity and ability to assimilate simple carbon compounds in deep subsurface environments is needed.

Previous studies of the Outokumpu scientific drill hole microbial community has concentrated on depths from 500 m and below [5],[6],[8], and only few studies exist that describe the microbial community in the upper parts of the Outokumpu subsurface [9],[10]. In addition, only little is presently known about the utilization of small carbon compounds by deep terrestrial subsurface microbial communities. Carbon is lost from biomass as carbon dioxide that is produced in different catabolic cellular processes, such as fermentation or oxidation of organic carbon if carbon dioxide is not recaptured. One of the main objectives of this study was thus to determine the proportion and identity of microorganisms responding to and harvesting inorganic carbon in the microbial community of groundwater in Outokumpu. In addition, we aimed to expand the knowledge of the deep subsurface microbial community in Outokumpu to include a fracture zone from a more moderate depth (180 m) than the previously studied deeper located fracture zones.

## 2. Materials and Method

### 2.1. Description of the sampling site

The Outokumpu Deep Drill Hole is situated in Eastern Finland in Paleoproterozoic, approximately 1.9 Ga old, bedrock. The lithology, hydrogeochemistry and gas composition of the drill hole water column and fracture zone fluids have been described previously [1]–[5],[11]. Shortly, the fracture zone at 180 m depth is situated in a metasedimentary rock sequence, predominated by mica schist.

### 2.2. Sampling and analytical methods for chemistry

The fracture zone at 180 m depth was isolated from the rest of the drill hole by inflatable packers as described in [10]. The isolated fracture zone was purged by pumping fluid via an air-tight poly acetate (PA) tube. In total around 23 m^3^ of water and 10 m^3^ of gas (at standard T and P) was drawn between the packers during the period between 5^th^ of May and 18^th^ of June 2012, with a typical pumping rate of 0.5 m^3^ of water per day [4]. Temperature, pH, electrical conductivity (EC), concentration of dissolved O_2_ and redox potential (Eh) of the pumped fluid was continuously monitored in a flow-through cell. Redox potential was measured using combined ORP electrode (Hamilton) with Ag/AgCl reference and values tested against a known reference solution. Measured values were converted to Eh-scale according to the operation manual of the electrode. Gas/water volume ratio was obtained from collected gas volume in a vessel under water and total water flow. Samples for geochemical analysis were taken from the pumped fluid usually twice a week. Samples for cation analysis (100 mL each) were filtered in the field using pore size of 0.45 µm and acidified with ultrapure HNO_3_ (0.5 mL per 100 mL of sample water) to prevent flocculation. Untreated samples of 250 mL each were used for anion analysis. Samples for sulfide analysis were fixed with 0.5 mL of 1 M NaOH and 0.5 mL of 1 M zinc acetate per 100 mL of water. Samples for Fe^2+^ were taken in glass bottles (Winkler) and fixed with concentrated HCl (4 mL per 100 mL of sample water). Gas samples were collected into inverted glass bottles (Schott, Duran Group, Main, Germany) under sample water and sealed with black butyl rubber stoppers. All samples were kept refrigerated until analyzed.

Cations were analyzed at Labtium Oy (Espoo, Finland): The concentrations of K, Mn, Zn, and I were determined using inductively coupled plasma mass spectrometer (ICP-MS, Perkin Elmer and Agilent Technologies) and S, Na, Mg, Fe_(tot)_ and Ca using inductively coupled plasma optical emission spectrometer (ICP-OES, Thermo Jarrell Ash Corp.), according the standard methods SFS-EN ISO 17294-2 and SFS-EN ISO 11885, respectively. Fe^2+^ was determined spectrophotometrically. Anions were determined by ion chromatography at Labtium Oy according the standard method SFS-EN ISO 10304-1 or at TVO Nuclear Services (Eurajoki, Finland) using a Dionex ICS-2000 device. Concentrations of sulfide were determined within 24 h from four parallel samples at Ramboll Analytics (Vantaa, Finland) using a spectrophotometer. Total and dissolved carbon was determined using a TOC analyzer at Labtium Oy according the standard method SFS-EN 1484, which is a pyrolytic method based on infra-red (IR) detection. End-point titration to pH 4.5 (standardized potentiometric method SFS 3005) was used to determine alkalinity. Gas compositions were determined by gas chromatography at Ramboll Analytics. Carbon speciation was calculated using PHREEQC software (USGS, 2014).

### 2.3. Sampling for microbiology and sample handling

Groundwater samples for microbiology were obtained on June 12–14, 2012 by pumping the fracture fluid via the PA tube directly in to an anaerobic chamber as described in [8]. Altogether 10 individual 50 mL samples were collected into sterile, acid washed 120 mL glass serum bottles sealed with butyl rubber stoppers (Bellco Glass Inc., NJ, USA) and aluminum crimp caps (Sigma, MO, USA) were collected for nano-scale secondary ion mass spectrometry (NanoSIMS) analysis, fluorescence microscopy, substrate induction (activation) and cell sorting, and cultivation. In addition, for the detection of transcription activation, four 2 L water samples were collected into sterile, anaerobic borosilicate glass bottles equipped with gas-tight butyl rubber septa. The biomass from two parallel 1 L water samples, one for DNA extraction and one for RNA extraction, was collected on 0.2 µm pore-size cellulose acetate filters (Corning) by vacuum suction as described by Purkamo et al. [8] directly in the field. The membrane filters were directly cut out of the filter funnels and fixed in dry ice. The rest of the water samples transported cooled (+4 °C) to the laboratory. In the laboratory, the water samples in the sealed bottles (both 50 mL and 1 L) were starved for 4 weeks by keeping the sampling bottles at +4 °C protected from light prior to the experiment.

### 2.4. Determination of cell numbers and proportion of viable microbial cells by epifluorescence microscopy

The total number of microbial cells in the groundwater at the time of sampling was determined by epifluorescence microscopy of 4′,6 diamidino-2-phenylindole dihydrochloride (DAPI) (Sigma, MO, USA) stained cells as previously described [7]. In addition, the percentage of intact microbial cells (i.e. microbial cells containing plasma membrane integrity) in the fracture fluid at the time of sampling and after 4 weeks of starvation was determined by LIVE/DEAD^®^ BacLight™ Bacterial Viability staining (Molecular Probes, Invitrogen, CA, USA) according to the manufacturer's instructions, followed by epifluorescence microscopy as previously described [11]. The analyses were done with two parallel fracture fluid samples of 1 mL or 2.5 mL. The microbial cells in the stained groundwater were collected on black GTPB Membrane filter (Millipore, Billercia, MA, USA) using a Millipore filtering unit (Millipore, Billercia, MA, USA). The average number of microbial cells mL^−1^ groundwater was calculated from 30 randomly chosen microscopic fields from each subsample, taking into account the volume (1 or 2.5 mL) of the sample collected on the filter and the total (283.53 mm^2^) and the active (0.014888 mm^2^) area of the polycarbonate filter and magnification (1000×).

### 2.5. Induction of respiration and transcription by addition of substrates

The activating effect of small carbon substrates on the microbial community was identified by the redox indicating dye 5-cyano-2,3,-ditolyl tetrazolium chloride (CTC, Polysciences Inc., PA, USA). Tetrazolium salts function as artificial electron acceptors that are reduced in metabolically active microbial cells by the components of the electron transport chain or by dehydrogenase activity to form brightly fluorescing formazan crystals in the cells. 50 mL sterile, acid washed glass serum bottles (Wheaton, NJ, USA) were flushed with sterile N_2_ gas for 30 min, each containing 0.55 mL 50 mM CTC dye, and sealed with sterile butyl rubber stoppers (Bellco Glass Inc., NJ, USA) and open top aluminum crimp caps (Sigma, MO, USA). Fracture fluid, after 4 weeks of starvation, was anaerobically aliquoted (5 mL) through the butyl rubber stoppers into the sealed infusion bottles using a N_2_ flushed sterile syringe and needle. The treatments used were: (1) no substrate additions to the fracture fluid, and addition of (2) sulfate, (3) CO_2_ and (4) CO_2_ + sulfate to fracture fluid samples. Sulfate was added through a 0.2 µm pore size syringe filter with an anaerobic N_2_-flushed syringe and needle through the rubber stoppers to the fracture fluid to a final concentration of 0.375 mmol/L. CO_2_ gas (20 mL, equaling a total of 0.9 mmoles, of which a maximum concentration of 0.18 mmol/L may be dissolved in the water sample) was added to the sealed infusion bottles using a syringe and needle pushed through the butyl rubber stopper. All gases were filter sterilized before injection by attaching a 0.2 µm pore size filter between the syringe and the needle. The sulphate solution (Na_2_SO_4_ 8.88 mg mL^−1^ in anaerobic MilliQ water stock solution, Sigma) was rendered anaerobic by N_2_ flush for 30 min. The samples were incubated together with the CTC dye for 6 h, according to the manufacturers recommendations, at +14 °C on a shaker (45 rpm), after which 0.5 mL glycerol-TE buffer was added to the samples for preservation at −80 °C according to the protocol by the Single Cell Genomics Center (https://scgc.bigelow.org/PDFs/Sample_cryopreservation_glyTE.pdf). Briefly the samples were divided into 1 mL aliquots in sterile cryo tubes and immediately frozen in liquid N_2_ and stored at −80 °C. A non-dyed reference sample was prepared and cryo-preserved in the same way as the dyed samples.

2 L batches of the stored groundwater were prepared for RNA extraction of the transcriptionally activated microbial community using the same substrates as described above. In addition, one 2 L batch of stored groundwater was used as baseline for the activation process. The experiment was performed in 2 L borosilicate bottles (Schott) equipped with butyl rubber stoppers and open-top screw caps. Anaerobic, sterile sulphate solution (Na_2_SO_4_) was added to a concentration of 0.375 mmol/L using a syringe and needle pushed through the butyl rubber stopper as described above. Carbon dioxide gas, 320 ml, was added with a sterile syringe and needle through the butyl rubber stopper as described above. The 2 L bottled were shaken vigorously by hand at the beginning of the incubation and kept at +14 °C for 2 h. After the incubation, the biomass from the groundwater samples was collected on 0.2 µm pore-size cellulose acetate bottle-top filters (Corning) by vacuum suction. The filters were cut out of the filter funnels using sterile scalpels, divided into two replicate samples of each treatment, representing two 1 L replicates, which were inserted into sterile, 50 mL screw-cap test tubes (Corning) and immediately frozen at −80 °C until RNA extraction.

### 2.6. Sorting of active cells by FACS

The CTC dyed and un-dyed fracture fluid aliquots were carefully thawed on ice prior to screening by flow cytometry (BD FACSaria flow cytometer, Becton Dickinson, NJ, USA). Samples were injected into a sterile phosphate buffered saline (PBS) flow stream and fluorescence was detected using a 655 nm long pass and 675/20 nm band pass filters. For excitation of CTC, an argon laser was used (488 nm). To detect the metabolically active cells, simultaneous measurements of forward light scatter (relative size), side light scatter (cell granularity), and CTC fluorescence emission were used, by setting the PMT voltage to 250, 250 and 500 volts, respectively. The side scatter threshold was set to 2000. To investigate the concentration of metabolically active cells, events were acquired over a time of 30 s with a flow rate of 100 µL min^−1^ from each sample. The fluorescence signal was plotted to the side scatter and analyzed with the BD FACSDiva™ 5.1 software (Becton Dickinson, NJ, USA). The CTC positive cells were gated comparing the fluorescence intensities of the dyed to the un-dyed fracture fluid sample. For downstream analysis and identification of the active microbial population, CTC positive cells were sorted into wells on sterile 8-strip 250 µL tubes, 10000 microbial cells/tube in approximately 100 µL solution. In addition, 10000 randomly selected cells were also collected from the un-dyed groundwater sample in order to determine the composition of the general community in the groundwater. Empty wells were left in between each sorted sample as negative controls. The sorted cells were immediately frozen on dry ice and stored at −80 °C until further analysed.

### 2.7. Nucleic acid extraction

#### 2.7.1. DNA extraction from sorted cells

All cell lysis and DNA amplification work was conducted in a UV light-treated laminar flow hood using filter-equipped, gamma-sterilized pipette tips. DNA was extracted from the sorted, active microbial populations by freeze-thaw cycles. The sorted cells were frozen (−25 °C) and heated for 10 min at 99 °C block temperature and at 105 °C lid temperature, in a MasterCycler Thermal cycler (Eppendorf, Hamburg, Germany) and re-frozen on a −25 °C close-fitting freeze block (Eppendorf, Hamburg, Germany) in a freezer for 30 min. This cycle was repeated 2 times, after which the released DNA was precipitated with 0.5 mL 94% ethanol. The released DNA was allowed to precipitate at −25 °C for 15 min after which the precipitates were collected by centrifugation +4 °C at 14000 rpm for 20 min in a table-top centrifuge (Eppendorf, Hamburg, Germany). The ethanol was decanted and the DNA pellet was allowed to dry at 37 °C in a heat block in a laminar flow hood. The DNA was resuspended in 10 µL molecular grade water. Negative reagents controls were treated parallel to the samples in order to control possible contamination of the samples by external sources.

#### 2.7.2. DNA and RNA extraction from groundwater samples

The filters with the collected microbial biomass were shortly thawed on ice before RNA extraction. The RNA was separately extracted from each half filter using the Mobio PowerWater RNA extraction kit (Mobio Laboratories, Inc., Carlsbad, CA, USA) according to the manufacturer's instructions. The RNA of each extraction was eluted into 100 µl of buffer PWR8 (Mobio Laboratories, Inc., Carlsbad, CA, USA). Negative control extractions were performed in parallel to the samples. The RNA extracts were tested for remnant DNA by performing PCR with general primers targeting the bacterial 16S rRNA gene. The primers used were the U968f and U1401r [12] using the Dynazyme II DNA polymerase as described in [13]. *E. coli* DNA was used as positive control for the PCR. No PCR product in the PCR reaction of the RNA extracts indicated that no residual DNA was present. The DNA from the groundwater from the day of sampling was extracted using the Mobio PowerSoil DNA extraction kit according to the manufacturer's instructions. The DNA was eluated in 100 µl buffer CS6.

### 2.8. Reverse transcription of RNA

The extracted RNA was reverse transcribed using the Superscript III First Strand Synthesis SuperMix (Invitrogen, Carlsbad, CA, USA) according to the manufacturer's instructions. First, 11.5 µL aliquots of RNA were incubated together with 250 ng random hexamers (Promega, WI, USA) and 0.83 mmol/L (final concentration) dNTP (Finnzymes, Espoo, Finland) at 65 °C for 5 min. Thereafter the reactions were cooled on ice for 1 min followed by the reverse transcription reaction when 4 µL 5× First strand buffer, 40 U DTT and 200 U Superscript III enzyme were added. The RNA was protected from degradation by addition of 40 U recombinant RNase inhibitor RNaseOut (Promega, WI, USA). The reactions were incubated at 25 °C for 5 min, 50 °C for 1 h and finally inactivated at 70 °C for 15 min.

### 2.9. Amplicon library preparation and sequence analysis

The bacterial and archaeal community composition of the sorted cell batches and the 1 L DNA and RNA samples were examined by high throughput amplicon sequencing using the Ion Torrent PGM platform as described in [7]. The amplification libraries were prepared by PCR from the DNA and cDNA samples. Bacterial 16S rRNA genes were targeted with primers S-D-Bact-0341-b-S-17/S-D-Bact-0785-a-A-21 [14], targeting the variable region V3–V4 of the 16S rDNA gene, and the archaeal 16S genes with primers S-D-Arch-0349-a-S-17/S-D-Arch-0787-a-A-20 [15], targeting the V4 region of the gene. The fungal communities were targeted with primers ITS1 and 58A2R flanking the internal transcribed spacer region 1 (ITS1) [16]. PCR amplifications were performed in parallel 25 µl reactions for every sample using the MyTaqTM Red Mix (Bioline, London, UK). Each reaction contained 20 pmol forward and reverse primers and 2 µL of template. The PCR program consisted of an initial denaturation step at 95 °C for 3 min, 40 cycles of 15 s at 95 °C, 15 s at 50 °C, and 15 s at 72 °C. A final elongation step of 30 s was performed at 72 °C. The PCR products were verified with agarose gel electrophoresis. Amplicons were sent for sequencing to Bioser at the University of Oulu (Finland) where the amplicons underwent purification and size selection before sequencing.

The sequence reads obtained from the Ion Torrent sequencing were subjected to quality control using the QIIME software version 1.9 as described in [7]. The sequences were grouped in to Operational Taxonomic Units (OTUs), following the open-reference OTU-picking protocol of QIIME and using the Silva version 128 database [17] for bacteria and archaea and the UNITE database version 6 [18] for fungi. Microbial community composition between the original groundwater, sorted cells and actively transcribing communities was tested by principal coordinates analysis (PCoA) using the Phyloseq package in R [19],[20].

The sequences were deposited in ENA under study number PRJEB22697.

### 2.10. Isolation of bacterial strains from fracture fluid

Bacterial strains were isolated on anaerobic Basal Mineral Medium [21] amended with 10 g L^−1^ NaCl to obtain pure cultures able to utilize carbonate as carbon source. The medium was composed of L^−1^ Na_2_HPO_4_·12 H_2_O, 9 g, KH_2_PO_4_, 1.5 g, NH_4_Cl, 1.0 g, MgSO_4_·7 H_2_O, 0.2 g, trace elements L^−1^ ZnSO_4_·7 H_2_O, 100 µg, MnCl_2_, 30 µg, H_3_BO_3_, 300 µg, CoCl_2_·6 H_2_O, 200 µg, CuCl_2_·2 H_2_O, 10 µg, NiCl_2_·6 H_2_O, 20 µg, Na_2_MoO_4_·2 H_2_O, 30 µg, ferric ammonium citrate 5 mg, CaCl_2_·2 H_2_O, 10 mg and 5 g NaHCO_3_. The medium was aliquoted in 10 mL portions into glass serum bottles (Wheaton, NJ, USA) and purged with filtered N_2_ gas for 1 h L^−1^ medium in order to render the medium anaerobic. The bottles were sealed with butyl rubber stoppers (Bellco Glass Inc., Vineland, NJ, USA) and open top aluminum crimp caps (Sigma, MO, USA) before autoclaving. The medium was solidified with 1.7% agar. Before the medium solidified 50 µL L-cystein hydrochloride reduced resazurine (10 µg L-cystein hydrochloride and 25 µg resazurin mL^−1^ stock solution) was added to the medium in order to maintain reducing conditions in the culture bottles. The resazurine functioned as oxygen indicator. After solidification, the medium was inoculated by anaerobically injecting 1 mL groundwater sample into the sealed medium-containing flasks through the butyl rubber septum using a sterile, N_2_ flushed syringe and needle. Bacterial cells in the water sample were allowed to attach to the agar surface for 30 min before the excess water was removed. Colonies were allowed to form for 10 days at +14 °C after which 10 colonies were picked for purification. The colonies were purified with three consecutive streak dilutions on individual agar plates after which DNA was extracted from individual colonies by suspending them in water (50 µL) and lysing the bacterial cells by heating in a heat block at 99 °C for 10 min. The bacterial strains were identified by 16S rRNA gene PCR and sequencing using the fD1 and U1401r primers [12],[22].

### 2.11. Nano-scale secondary ion mass spectroscopy (NanoSIMS)

Aliquots (50 mL) of groundwater were amended with anaerobic, filter sterilized ^13^C sodium carbonate or ^12^C sodium carbonate (Sigma, MO, USA) as to a final concentration of 4 mmol/L, by injection through the butyl rubber stopper as described above. The samples were incubated at +14 °C for 60 days. In addition, pure cultured bacterial strains (described above) were re-grown in Basal Mineral Medium supplemented with ^13^C or ^12^C sodium carbonate (Sigma, MO, USA) to a final concentration of 4 mmol/L for 2 weeks. Subsamples of the prepared groundwater samples and the pure cultures were collected on membrane filters pre-coated with Au/Pt (30 nm, 208 HR High Resolution Sputter Coater, Cressington Scientific Instruments Inc, Cranberry, PA, USA) for scanning electron microscopy (SEM) and nano-scale secondary ion mass spectrometry (NanoSIMS). The preparations were fixed in phosphate (0.1 M, pH 7.2) buffered 2.5% glutaraldehyde at +4 °C for 20 h, and rinsed with phosphate buffer three times. Dehydration was carried out with an ethanol series from 30% to 50% to 70% to 80% to 96% and absolute, followed by hexamethyldisilazane (Fluka, Buchs, Switzerland). The samples were examined with a Hitachi S-4800 FESEM (Tokyo, Japan) operated at 1 kV and with NanoSIMS (Cameca NanoSIMS 50 L, Gennevilliers Cedex, France) at the Lehrstuhl für Bodenkunde (TU Munich, Germany). A fine focused Cs^+^ primary ion beam (about 1 pA) was used to produce secondary ions at a lateral resolution of about 100 nm. The instrument was tuned for a mass resolving power eligible to distinguish between ^12^C^1^H and ^13^C. The ^12^C^−^, ^13^C^−^ and ^12^C^14^N^−^ secondary ions were simultaneously collected in imaging mode using electron multipliers with a set dead time of 44 ns. Raster images of 13 × 13 µm^2^ and 15 × 15 µm^2^, 256 × 256 pixel were recorded with 30 ms dwell time. The instrument was checked regularly before measurements using a Si engraved reference sample. The ^12^C^14^N^−^ images were used for the ROI selection as these best represented the bacterial cells. The cumulative counts of every ROI were used to calculate the ^13^C^−^/^12^C^−^ ratio for every bacterial cell, which are shown as box plots illustrating natural abundance versus labelled bacterial cells. The background natural abundance value was obtained from the non-labelled bacterial cell ROI values. All analyses including the calculation of the ^13^C^−^/^12^C^−^ ratio images were processed using the Cameca WinImage Software.

### 2.12. Sequence analysis and phylogeny of the pure cultured strains

The obtained sequences were imported into Geneious Pro (version 6.0.1, Biomatters Ltd., Auckland, New Zealand) and manually checked, assembled and edited before being subjected to phylogenetic analysis. The sequences were compared with the blastn tool in Geneious Pro to the NCBI nucleotide database. The closest matching sequences, relevant reference sequences and sequences of suitable type species were included in the phylogenetic analyses. The bacterial 16S rRNA gene sequences were aligned using Muscle in Geneious Pro and the alignment was edited manually. A maximum likelihood cladogram was calculated for each domain using PhyML [23] with the Jukes-Cantor substitution model [24]. Bootstrap support for the branches was calculated with 1000 random repeats. The sequences were submitted to Genebank under accession numbers KP192167–KP192240.

## 3. Results

### 3.1. Physicochemical parameters

Fracture fluid was pumped from 180 m depth of the Outokumpu Deep Scientific Drill Hole to investigate the environmental characteristics and the microbial populations present in this habitat. During the pumping, the pH decreased from 11 to 8.5, indicating that the drill hole water, which has been disturbed by the alkaline effect of concrete casting of the uppermost 22 m of the drill hole, was effectively removed by the time of microbiological sampling. The fracture fluid at 180 m depth is brackish (Na-Ca-Cl type) with total dissolved solids (TDS) around 6 g L^−1^ (Table 1). Conditions were reducing with dissolved O_2_ below the limit of detection in the on-line measurements, Eh approximately −0.1 V and iron occurring as Fe^2+^. Gas/water ratio of discharging fluid was approximately 0.45 L L^−1^. Gas/water ratios determined from pressurized total fluid samples in the lab gave comparable results between 0.425 and 0.440. Dissolved gas phase comprised mostly of methane (up to 76 vol-%) and nitrogen (up to 22 vol-%), with minor amounts of He, Ar, ethane and propane. Some O_2_ was detected in the laboratory analyses of gas samples but is likely due to minor contamination from air during sampling and/or analysis. H_2_ was occasionally detected, but generally remained below the detection limit of 0.003 vol-%. Likewise, CO_2_ was not present in detectable amounts. Very little inorganic carbon, of which 81% (0.23 mmol/L) was HCO_3_^−^, was found. The total amount of carbonic acid anions (HCO_3_^−^, CO_3_^2−^) was low as indicated by the low alkalinity (∼0.3 mmol/L) and low concentration of dissolved inorganic carbon (0.21 mmol/L). Consequently, the dominant form of carbon in the fracture fluid was methane (i.e., about 15 mM or 240 mg L^−1^). Low concentrations of both sulfate (0.007 mmol/L) and sulfide (0.0018 mmol/L) were found.

**Table 1.** Geochemical characteristics of the groundwater from the isolated fracture at 180 m depth in the Outokumpu Deep Drill Hole. Groundwater pH, Eh, EC and dissolved oxygen were measured continuously in a flow-through cell. Dissolved gas phase composition analysed for gas phase extracted from water (gas to water ratio 0.45).

Measurement	Value	Date
pH	8.5	13.6.2012
Eh	−95 mV	13.6.2012
EC	1035 mS/m	13.6.2012
O_2_	0.0 mg/L	13.6.2012
Alkalinity	0.31 mmol/L	13.6.2012
O_2_	0.37 vol-%	13.6.2012
N_2_	21 vol-%	13.6.2012
CO_2_	<0.003 vol-%	13.6.2012
CH_4_	74 vol-%	13.6.2012
H_2_	<0.003 vol-%	13.6.2012
C_2_H_6_	0.86 vol-%	13.6.2012
C_3_H_8_	0.025 vol-%	13.6.2012
He	1.5 vol-%	13.6.2012
Ar	0.27 vol-%	13.6.2012
TOC	0.48 mmol/L	6.6.2012
DOC	0.45 mmol/L	6.6.2012
TIC	0.23 mmol/L	6.6.2012
DIC	0.21 mmol/L	6.6.2012
SO_4_	0.7 mg/L	5.6.2012
Sulfide	0.06 mg/L	6.6.2012
NO_3_	<20 mg/L	13.6.2012
Br	23 mg/L	13.6.2012
Cl	3280 mg/L	13.6.2012
I	300 mg/L	13.6.2012
F	0.2 mg/L	5.6.2012
S	1.27 mg/L	13.6.2012
Na	1070 mg/L	13.6.2012
Mg	16.7 mg/L	13.6.2012
Fe(tot)	0.34 mg/L	13.6.2012
Fe^2+^	0.49 mg/L	6.6.2012
Ca	1060 mg/L	13.6.2012
Zn	46.3 µg/L	13.6.2012
Mn	133 µg/L	13.6.2012
K	13.7 mg/L	13.6.2012

### 3.2. Microbial cell numbers and proportion of active respiring cells

**Figure 1.**
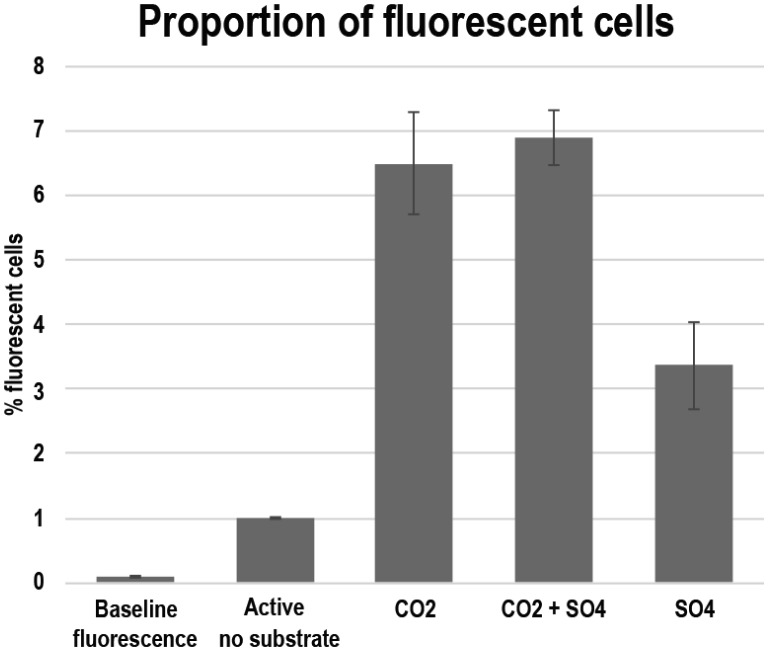
Relative amount of metabolically active fluorescent cells as determined by CTC staining and FACS. The average values were calculated from three parallel measurement and the error bars indicate standard error of mean.

**Figure 2.**
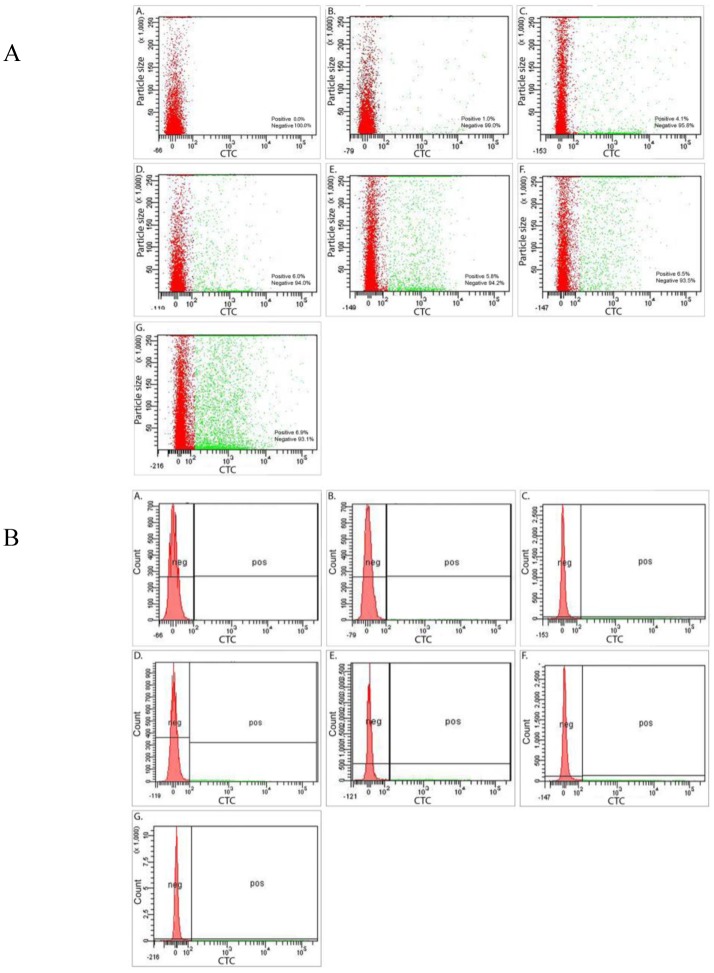
A: Forward scatter (showing the relative size of the particles) vs. fluorescence plots of the microbial population of the (A) undyed sample, (B) CTC dyed sample without addition of substrates, and CTC dyed samples treaded with (C) sulfate, (D) methane, (E) methane + sulfate, (F) CO_2_ and (G) CO_2_ + sulfate amended samples. The red dots describe the nonfluorescent i.e. inactive population, the green dots the activated fluorescent population. B: The defined sorting gates for the microbial population, A–G as in Figure 2A. The red histograms indicate the inactive (negative) population and the green histograms the active fluorescent (positive) population.

The concentration of microbial cells as determined by DAPI staining and microscopy was 3.0 × 10^5^ mL^−1^ (std 8.8 × 10^4^), of which 87% (2.6 × 10^5^ mL^−1^) were viable as determined with the live/dead staining. After 4 weeks of storage in order to deplete carbon and nutrients, the cell number had slightly increased to 3.6 × 10^5^ mL^−1^ (std 5.6 × 10^4^), and all cells appeared viable as determined with the live/dead staining. The number of microbial cells determined by FACS in the redox stained samples was 4.2 × 10^5^ mL^−1^ (std 1.9 × 10^4^), 5.3 × 10^5^ mL^−1^ (std 4.6 × 10^4^) and 4.8 × 10^5^ mL^−1^ (std 8.9 × 10^4^) in the samples that had been incubated together with CO_2_, CO_2_ + SO_4_ and SO_4_, respectively. In contrast to the viability staining, the redox indicating CTC staining showed that only approximately 1% of the measured events (equivalent to microbial cells) fluoresced and were detected by FACS (Figure 1 and Figure 2). The response of the inactive microbial cells to methane, as the most common carbon compound on Outokumpu deep subsurface, and CO_2_, as substrate for autotrophic microorganisms, was tested with CTC. In addition, SO_4_ was added as terminal electron acceptor since it is the most common one detected in Outokumpu, although the concentration is low. The CO_2_ with or without SO_4_ had the greatest activating effect on the cells. The CO_2_ + SO_4_ treatment increased the relative abundance of active cells to 6.9% and CO_2_ alone to 6.5%. CH_4_ and CH_4_ + SO_4_ increased the proportion of activated cells to 6% and 5.9%, respectively. Sulfate alone increased the proportion of activated cells to 3.4% of the microbial cells.

### 3.3. Characterization of the general and actively respiring fraction of the microbial community

The composition of the microbial community in the untreated groundwater and the actively respiring community was studied from 10000 cells collected from each treatment with 16S rRNA gene PCR followed by high throughput amplicon sequencing of the bacterial and archaeal 16S rRNA gene and fungal ITS1 region. In addition, the bacterial and archaeal 16S rRNA and rRNA gene profiles and fungal ITS1 profiles were characterized from the DNA and RNA from parallel 500 mL water samples of the original groundwater as well as RNA from parallel 500 mL water after storage and addition of CO_2_ and/or SO_4_ with the same high throughput sequencing technique as above.

Between 2701 and 12476 bacterial, 1807 and 12270 archaeal and 401 and 20352 fungal sequence reads were obtained from each DNA and RNA fraction of the groundwater samples (Table 2). Nevertheless, the RNA fraction of the groundwater samples that had received no substrate additions did not yield any archaeal 16S rRNA or fungal ITS transcripts. ITS transcripts were also very scarce in the RNA fraction of the groundwater collected directly in the field and were detected from only one of the parallel samples. The number of sequence reads obtained from the DNA of the sorted cells varied between 1362 and 6811 and belonged only to bacteria. No archaeal 16S rRNA or fungal ITS transcripts were detected in the sorted cells. The number of bacterial OTUs detected was high, ranging from 530 to 2030 per sample, while the archaeal OTUs ranged from 225 to 793. The number of fungal ITS OTUs was high in the DNA fraction of the groundwater (532 to 637 OTUs) but in the RNA fractions the OTU numbers stayed below 70. According to the Chao1 richness estimate, between 30 and 53% of the estimated number of bacterial OTUs present in the samples were detected and 38–60% of the archaeal OTUs. The fungal diversity was lower than that of the bacterial and archaeal, and between 87 and 99% of the predicted fungal ITS OTUs were detected. The Shannon diversity index calculated based on the number and abundance of detected OTUs was highest for the bacterial community, ranging between 4.9 and 10.0 (Table 2). For the archaeal community, the Shannon index ranged between 3.0 and 4.2 and the fungal one between 1.1 and 5.6.

**Figure 3.**
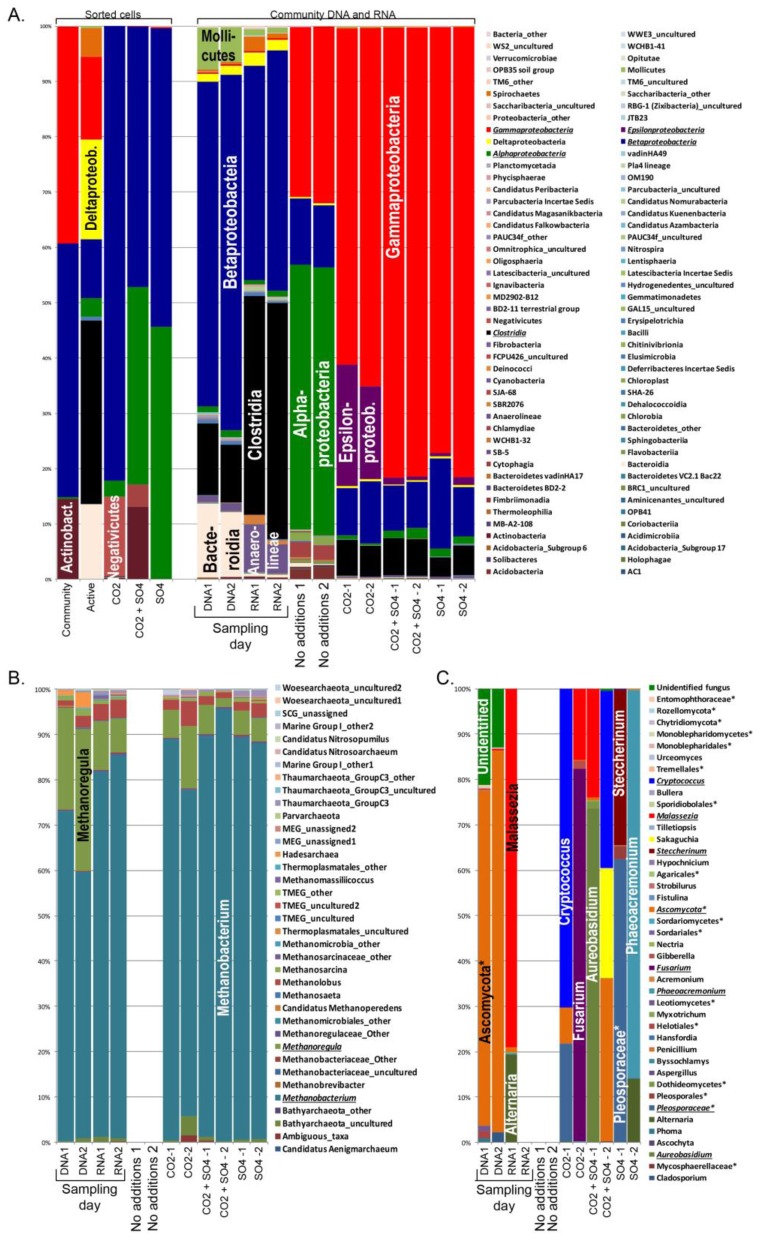
The relative abundances of (A) bacterial classes, (B) archaeal and (C) fungal genera identified from the sorted cells and the DNA and RNA fraction of the original fracture zone water and from the RNA fraction of the treated water. Major microbial taxa are indicated with italics and underline. * indicates unidentified taxon of a specific group.

The original fracture zone bacterial community consisted mostly of Betaproteobacteria (58.7–64.3%) with Clostridia (10.5–13%), Bacteroidia (11.6–13.4%) and Mollicutes (6.3–7.6%) as minor groups (Figure 3A). The same bacterial groups were detected in the RNA fraction, but Clostridia contributed with 39.6–42.6% while the proportion of Betaproteobacteria was 38.7–43.4%. Over the time of storage of the groundwater, the relative abundance of 16S rRNA of alphaproteobacteria had increased from approximately 1–3% in the original groundwater to 47.8–48.4% in the stored groundwater at the time of the experiment. The relative abundance of betaprotobacterial 16S rRNA sequences had decreased to 11.3–12.0% and gammaproteobacterial 16S rRNA sequences from below 1% of the sequence reads to 30.7–31.8% of the sequence reads. After addition of CO_2_ the relative abundance of gammaproteobacterial 16S rRNA sequence reads increased to 60.7–64.7%, the betaproteobacterial 16S rRNA sequence reads remained at 8.5–11.2%, while the transcription of epsilonproteobacterial 16S rRNA genes increased from negligent to 16.8–22.0%. The majority of the detected gammaproteobacterial sequences belonged to the *Pseudomonas* and the betaproteobacterial sequences to *Hydrogenophaga*. Of the epsilonproteobacterial sequences, one third belonged to *Sulfuricurvum* and the rest to *Sulfurimonas*. Clostridial 16S rRNA sequences were not detected in the untreated stored water, but after addition of CO_2_ the relative abundance of clostridial 16S rRNA sequences increased to 5.7–6.5%. The most abundant clostridial sequences belonged to Desulfosporosinus and an uncultured Peptococcaceae genus. The bacterial 16S rRNA profile detected in the water samples that had received CO_2_ + SO_4_ or only SO_4_ were very similar with 77.0–81.3% gammaproteobacteria, 8.2–16.3% Betaproteobacteria, 1.3–1.9% Alphaproteobacteria and 3.5–6.6% Clostridia.

The bacterial 16S rRNA and rRNA gene profiles obtained from the sorted cell fractions differed from the profile of the extracted nucleic acids (Figure 3A). The structure of the bacterial community in the groundwater consisted of 39.2% Gammaproteobacteria, 45.8% Betaproteobacteria and 14.5% Actinobacteria and the active bacterial cells belonged to Spirochaetes (5.1%), Gammaproteobacteria (14.9%), Deltaproteobacteria (18.1%), Betaproteobacteria (10.6%), Alphaproteobacteria (3.3%), Clostridia (33.2%) and Bacteroidia (13.6%). CO_2_ activated especially Betaproteobacteria (82.2%) and Negativicutes (14.4%) and CO_2_ + SO_4_ or only SO_4_ activated Betaproteobacteria (47.0–54.1%) and Alphaproteobacteria (35.7–45.5%). In addition, CO_2_ + SO_4_ also activated Actinobacteria (13.3%).

**Figure 4.**
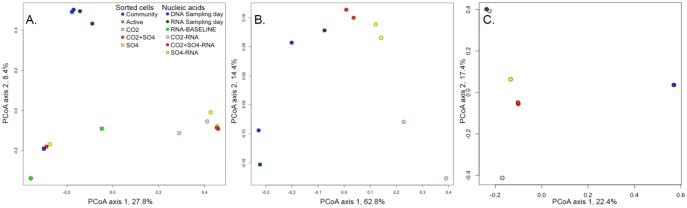
PCoA analysis calculated based on Bray-Curtis dissimilarity of the (A) bacterial, (B) archaeal and (C) fungal community profiles identified by high throughput amplicon sequencing of sorted cells (squares) and DNA or RNA extracted from the fracture zone and induced groundwater (circles).

The archaeal profiles in the original groundwater and in the different treatments were similar to each other (Figure 3B). The majority of the archaeal community detected from both the DNA and RNA fractions of the original water consisted of members of the genus *Methanobacteria*, contributing with 58.6–84.3% of the sequence reads (Figure 4B). In addition, *Methanoregula* (7.4–31.5%) and *Methanolobus* (1.1–4.0%) were abundant in the original groundwater. Hadesarchaea were present in the DNA fraction (1.2–3.4%) but below 1% of the archaeal 16S rRNA sequences in the RNA fraction belonged to this group. In addition, small amounts of Bathyarchaeota, Woesearchaeota and Thaumarchaeota were detected. No archaeal 16S rRNA sequences were obtained from the stored, untreated water. However, after addition of CO_2_ and/or SO_4_ archaea were again detected as the transcription of the archaeal 16S rRNA resumed and the 16S rRNA profiled were similar to that of the groundwater at the time of sampling, with *Methanobacterium* as the dominating archaeal group. No archaea were detected from the sorted cells.

The fungal consortia in the original groundwater consisted mainly of an unidentified clade within the Ascomycota (73.7–100% of the ITS sequence reads) (Figure 3C). In addition, an unidentified fungal group was detected in the DNA fraction (13.0–21.3%) and in the other RNA sample of the original groundwater the transcripts detected belonged to *Malassezia* (79.0%) and *Alternaria* (19.5%). No ITS transcripts were detected after storage of the groundwater or in the second RNA sample from the original groundwater. However, addition of CO_2_ and/or SO_4_ increased the transcription rate. The fungal ITS sequence profile differed a lot between the different treatments and between the replicate samples of the treatments. After CO_2_ addition, ITS sequences belonging to *Cryptococcus* (70.2%), unidentified Ascomycota (8.1%) and unidentified Pleosporaceae (21.4%) were detected in one of the replicate samples while the other contained *Fusarium* (82.1%) and *Malassezia* (15.8%). CO_2_ + SO_4_ activated *Aureobasidium* (73.5%) and *Malassezia* (23.9%) in one replicate sample and *Cryptococcus* (39.1%) *Sakaguchia* (24.1%) and unidentified Ascomycetes (40.0%) in the other. SO_4_ activated unidentified Pleosporaceae (62.2%) and Steccherinum (34.4%) in one sample and *Phaeoacremonium* (85.6%) and *Alternaria* (14.0%) in the other. As the archaea, no fungal ITS sequences were obtained from the sorted cells.

The clustering of the samples based on the detected community composition reflected the community composition seen in the taxonomic analyses (Figure 3 and Figure 4). In the Principal Coordinates Analysis (PCoA), the bacterial communities detected in the original groundwater separated these samples from the rest in the upper left quadrant of the plot. The active community in the sample water after storage (RNA-baseline) were located in the opposite left corner of the plot, and the active community detected after addition of substrates fell to the right side of the plot, indicating that a different bacterial population was activated by the added substrates compared to that, which was active before adding the substrates. The composition of active cells that were sorted by FACS before DNA extraction and sequencing fell separately from the rest of the samples. The result indicates that the bacterial cells that increase respiration (as detected by CTC fluorescence) may not increase their transcription of ribosomal genes and that the rate of transcription of ribosomal genes may be triggered by other metabolic activities than respiration. The archaeal community detected in the original groundwater and the activated samples were very similar. The samples that had received SO_4_ or CO_2_ + SO_4_ fell close to each other on the PCoA plot together with samples from the original groundwater, while the samples that had received only CO_2_ were located in the opposite direction (Figure 4B). The distribution of the fungal communities on the PCoA plot reflected the same variability as seen in the taxonomic analyses (Figure 4C).

**Table 2.** Alpha-diversity metrics based on the absolute number of sequence reads belonging to bacteria, archaea or fungi, respectively.

	Bacteria				Archaea				Fungi		
	Number of sequences	Number of OTUs	Chao1	Shannon H'	Number of sequences	Number of OTUs	Chao1	Shannon H'	Number of OTUs	Chao1	Shannon H'
DNA sampling day	10541–12311	1166–1266	2207–2813	5.2–5.7	8341–11081	652–793	1282–1381	4.1–4.2	532–677	600–707	4.9–5.6
RNA sampling day	7099–12467	886–1278	1954–2654	5.6–5.9	6039–12270	570–875	1005–1481	4	14	15	1.7
No additions	2701–3247	538–633	1376–1733	6.1	n.d.	n.d	n.d.	n.d.	n.d.	n.d.	n.d.
CO_2_	4562–12420	607–1226	1202–2821	5.0–5.4	1807–3226	225–398	588–922	3.0–4.1	24–26	26–28	1.4
CO_2_ + SO_4_	9882–10863	1093–1200	2509–2552	4.9–5.0	4688–4718	434–492	870–1022	3.0–3.5	32–67	33–67	1.9–2.3
SO_4_	9957–12410	1077–1231	2193–2952	4.9–5.0	3840–3916	397–451	762–932	3.3–3.6	26–44	27–44	1.1–1.7
*Community	3272	1475	5619	8.6	n.d.						
*Active	6811	1248	2818	6.4	n.d.						
*CO_2_	1361	925	3178	9.2	n.d.						
*CO_2_ + SO_4_	4317	2030	5680	9.9	n.d.						
*SO_4_	4505	1900	4202	10	n.d.						

* sorted cells, n.d. not deteted.

### 3.4. Identification of pure cultured bacterial strains

**Figure 5.**
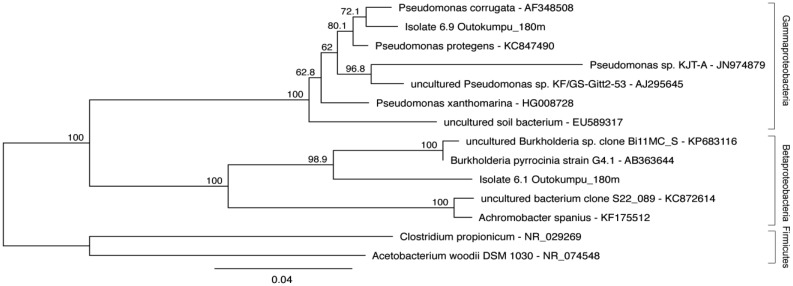
Phylogenetic tree placing the two isolated bacterial strains tested by NanoSIMSs with the *Pseudomonas* sp. and the *Burkholderia* sp. Bootstrap support for nodes was calculated from 1000 random repeats and are shown for nodes with >50% support.

In order to test the incorporation of inorganic carbon into bacterial cell structures of indigenous groundwater bacteria colonies from the groundwater were isolated in autotrophic and anoxic culture conditions. Sample water was inoculated into sealed anaerobic infusion bottles containing Basal Mineral Media containing Na-carbonate. Colonies formed on the anaerobic agar were identified by 16S rRNA gene sequencing. The bacterial strains obtained belonged to *Betaproteobacteria* (three strains) and *Gammaproteobacteria* (1 strain) or remained unidentified because the cultures ceased to grow. One betaproteobacterial strain belonging to the genus *Burkholderia* (isolate 6.1) and one gammaproteobacterial strain belonging to the *Pseudomonas* (isolate 6.9) (Figure 5) were included in the NanoSIMS experiment. SEM analysis showed that the cells of both isolates were approximately 0.2 × 2 µm (isolate 6.1) and 0.25 × 1.5 µm (isolate 6.9) rods.

### 3.5. Enrichment of ^13^C carbon in microbial cells

**Figure 6.**
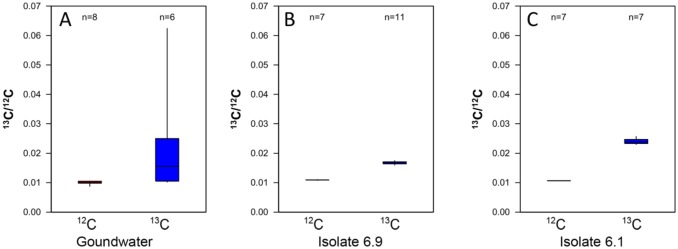
Box plots displaying the mean ^13^C enrichment of the individual microbial cells detected by NanoSIMS in the (A) fracture fluid community enrichment, (B) isolate 6.1 and (C) isolate 6.9 cultures. The left box displays the background ^13^C enrichment of the ^12^C carbonate treated cells and the right box the ^13^C enrichment of the ^13^C carbonate treated cells. The n-value in the plots indicated the number of individual cells detected in the different treatments from which the enrichment ratio has been calculated.

**Figure 7.**
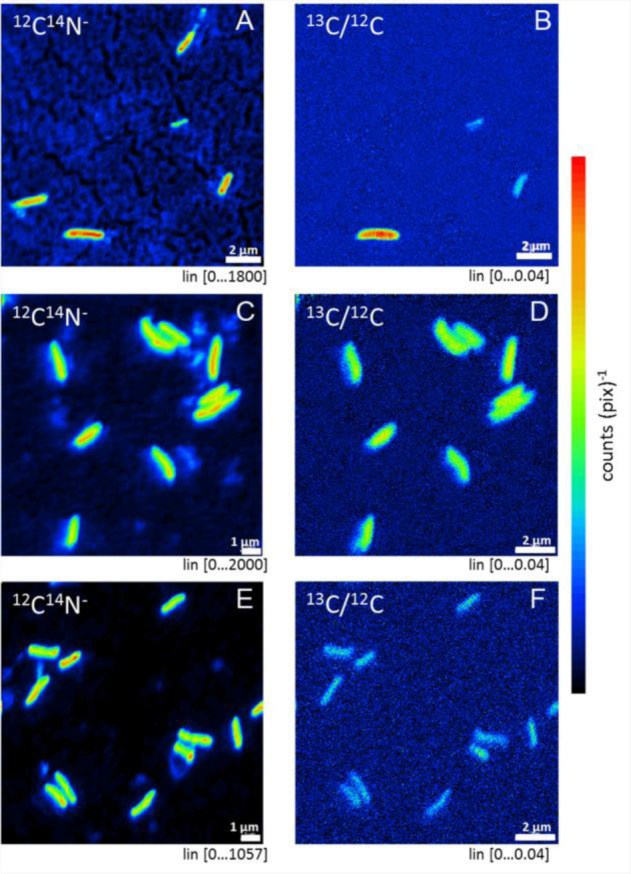
NanoSIMS images of the (A–B) fracture fluid community, (C–D) *Pseudomonas* sp. isolate 6.9 and (E–F) *Burkholderia* sp. isolate 6.1. The left column (A, C, E) displays measurements of ion masses ^12^C^14^N, the right column (B, D, F) the enrichment of ^13^C in the bacterial cells.

The incorporation of C from carbonate in to microbial cells over 2 weeks for the pure cultures and 2 months for the groundwater community was examined with NanoSIMS. The ^13^C enrichment in the microbial cells was tested by using control samples that had been treated with ^12^C carbonate. In the ^12^C carbonate control samples the ^13^C/^12^C ratio was approximately 0.01 in all detected cells (Figure 6, ^12^C treatment). For the fracture fluid samples treated with ^13^C carbonate as carbon source, 50% of the detected microbial cells (n = 6) revealed by the ^12^C^14^N-signal showed enrichment in ^13^C with a mean ^13^C/^12^C ratio of up to 0.024 (Figure 6, Figure 7A, Figure 7B). However, when examining the pure cultures belonging to *Pseudomonas* sp. (Isolate 6.9) and *Burkholderia* sp. (Isolate 6.1) (Figure 5), all observed microbial cells had incorporated ^13^C (Figure 7 C–F) and the ^13^C/^12^C mean ratio was 0.016 and 0.023, respectively (Figure 6B and Figure 6C).

## 4. Discussion

Outokumpu Deep Drill Hole with a total depth of 2516 m has been under intensive investigations since its drilling in 2004–2005 [2]. The deep subsurface at Outokumpu presents a wide diversity of microorganisms with metabolically diverse communities [1],[5],[9],[10]. The use of carbon sources and electron acceptors by the microbial communities as well as their activity are some of the major questions in deep subsurface research. Here we have addressed them by using substrate-induced activation of the microorganisms, sorting of activated cells, sequencing of 16S rRNA and ITS1 transcripts and NanoSIMS.

The metabolic activity of microorganisms in deep subsurface environments is extremely low [26],[27]. This may be due to specific limiting factors, such as lack of electron donors or acceptors in the environment. In the deep subsurface of Outokumpu the most abundant carbon source is methane, which is constantly released from deep fracture zones [3],[4]. In addition, the sulfur cycle is tightly connected to the carbon cycle, i.e. carbon compounds are oxidized through sulfate reduction. CO_2_ assimilation by chemoorganotrophic microorganisms has been shown to be a major process in the Outokumpu deep subsurface environment with autotrophic pathways becoming more common only at greater depths [1]. We have also previously demonstrated that the microbial community in the fracture zone at 500 m depth in Outokumpu were substantially revived by methanol and methane and that sulfate as electron acceptor together with these carbon sources greatly increased the proportion of metabolically active microbial cells in the fracture fluid [6]. In addition, we showed that methane and methanol activates the transcription of 16S rRNA genes of different bacteria and archaea in the fracture zone at 180 m compared to that at 500 m depth [7].

The abundance of microbial cells as well as their viability (i.e. membrane integrity) and respirational activity can be determined using different fluorescent dyes (e.g. [27],[28]). For example, 4′,6 diamidino-2-phenylindole dihydrochloride (DAPI) is used to identify DNA-containing microbial cells for total cell counts, the live/dead staining is used to confirm bacterial membrane integrity, and 5-cyano-2,3-ditolyl tetrazolium chloride (CTC) is used to evaluate respiratory activity in bacterial cells. In the present study, we used the live/dead staining to determine the proportion of intact microbial cells in Outokumpu fracture fluid from the depth of 180 m, and CTC to detect respiring microbial cells in the fluids. CTC has previously been used for detection of respiratory active microbial cells for example in sediments [29], in the water column of Lake Kinneret [27] and an eutrophied river [30]. CTC stained fluorescing cells have also been shown to be better compatible with flow cytometry than with microscopy because the flow cytometer may detect even lightly fluorescing active cells [31]. Here, when the live/dead stained and CTC stained samples were compared, we found a great difference in the proportion of intact cells compared to the respiratory active cells in the untreated fracture fluid. This indicates that most of the bacterial cells were not respiring, but may use for example fermentation as means of energy generation, or that their level of respiration is too low to be detected by CTC. After addition of substrates to the groundwater samples, the relative abundance of respiratory active microbial cells increased as detected by reduction of CTC to fluorescent formazan in the cells and subsequent detection by FACS. This has been reported before with e.g. *Vibrio cholerae* cells in freshwater, which during prolonged starvation remained viable according to the live/dead staining, but lost cultivability over time [32]. Nevertheless, Créach et al. [33] showed that cultures of respiring *E. coli* still reduced small amounts of CTC in the lag phase of the culture although the CO_2_ production had ceased indicating that the cells could maintain enough energy for CTC reduction although the respiratory activity had stopped. Sieracki et al. [31] also showed that some microbial cells may transport CTC through their plasma membranes, but do not reduce it and would thus go undetected in this assay.

In contrast to Rajala et al. [6] and possibly due to the different method of detection, or higher original number of viable microorganisms in untreated groundwater, the number of microbial cells activated by substrates in this study was low, only around 7%, at the most. This is an indication that only a limited part of the microbial community in the fracture fluid at 180 m depth in Outokumpu utilizes carbon dioxide or carbonate. Similar results were previously obtained using methane and methanol, where the added carbon sources only activated a small part of the microbial cells at 180 m depth in Outokumpu, while much higher activation was detected in the fracture zone at 500 m depth [7]. Nevertheless, the archaeal 16S rRNA gene analyses (Figure 3B) indicate that the methanogenic population consist of CO_2_ + H_2_ utilizing methanogens. This result also agrees Kietäväinen et al. [4] who observed that isotopic fractionation between methane and water at 180 m is consistent with methanogenesis through CO_2_ reduction (or possibly some other mechanisms in which all hydrogen is derived from water, such as in the hypothetic case of methanogenesis from graphite and water) rather than acetate fermentation.

The respiratory active bacterial population differed greatly from the major bacterial groups present in the untreated fracture fluid. Interestingly, *Clostridia* have previously been shown to constitute only a minority in the upper groundwater of Outokumpu drill hole [5],[9],[10] and in the present study they failed detection from the 10000 sorted cells from the general microbial community. Nevertheless, in the active population harvested from samples that had not received any additional substrates the *Clostridia* constituted one of the major bacterial groups. This was also the situation when examining the RNA fraction of the groundwater on the day of sampling. However, in the substrate-amended samples the Clostridia were detected from the RNA fraction of the water samples that had received substrates, but not in the control water that had not received any amendments.

The DNA based methods reveal the microorganisms that are present (also dormant or dead cells and extracellular DNA), while the RNA based approach detects living and active microorganisms by their ribosomal RNA domains and mRNA transcripts. Previous studies of the Finnish deep biosphere have shown that the microbial community profile detected using 16S rRNA genes and ITS regions may differ significantly from the profile when 16S rRNA and ITS transcripts are targeted [34],[35],[36]. It has been reported that the turnover rate of extracellular DNA in marine water may be as low as 10 h, while in sediments it may take up to 29–93 days for the extracellular DNA to degrade [37]. In groundwater the turnover rate of DNA is not known. In addition, the activation of microbial processes, such as the transcription of specific genes or the production of ribosomes cannot be discerned, and therefore we studied the active and activated proportion of the microbial communities by specifically targeting the RNA fraction and using the DNA fraction in the original fracture water only as reference.

The most abundantly detected group of actively transcribing bacteria in Outokumpu bedrock fracture fluid at 180 m depth was the *Pseudomonas*, which dominated the 16S rRNA profiles in the amended water samples and were also a major group in the untreated control water. Nevertheless, 16S rRNA gene sequences of *Pseudomonas* bacteria have not been detected in the groundwater of Outokumpu at any depth before [5],[8],[10], and they were clearly below the limit of detection in the fracture zone water on the day of sampling. Nevertheless, Purkamo et al. [1] found a low number of *Pseudomonas*-like nitrate reductase (*nar*G) genes in Outokumpu groundwater specifically detected by *nar*G targeted PCR. Additionally, Rajala et al. [6] demonstrated that transcription of *Pseudomonas*-like *nar*G genes was activated when methane and methanol were available. Our results indicate that the *Pseudomonas* constitutes minority in the Outokumpu deep bedrock environment, although they have readily been detected in other Fennoscandian deep subsurface studies [13],[38]. These bacteria appear to have the ability to rapidly respond to changing environmental conditions. This ability may have great effects on e.g. storage of hazardous waste in deep geological repositories.

Betaproteobacteria were one of the bacterial groups detected in the total bacterial community in the starved, untreated water, but were also metabolically induced by especially CO_2_. Betaproteobacteria, such as the autotrophic, hydrogen-oxidizing *Hydrogenophaga* sp., have previously been shown to be one of the major bacterial groups in the upper parts of the Outokumpu deep drill hole and it has been hypothesized that they may play an important role in autotrophic carbon fixation processes in this environment [10]. Here, *Hydrogenophaga* were not detected in the sorted total microbial community from the fracture fluid at 180 m depth, nor as a group increasing their respiration in response to the addition of CO_2_ as carbon substrate. Instead the Betaproteobacteria detected belonged to *Ralstonia* sp. Nevertheless, both 16S rRNA genes and transcripts were abundant in the DNA and RNA fractions of the groundwater and treated water samples indicating that these bacteria still respond to the added substrates. These bacteria may have potentially important roles in capturing CO_2_ released in fermentation processes and assimilate it into biomolecules, which can be used by the rest of the microbial community.

The archaeal lineages detected from the RNA fraction of the water samples treated with CO_2_ or CO_2_ + SO_4_ were the same groups as were activated by the addition of methane or methanol to water from the 180 m fracture zone in our previous study [7]. *Methanobacterium* was the dominating archaeal type found in any of the samples at 180 m and it has been detected from this depth previously [34]. *Methanoregula* accounted for the second largest group of sequences, and also belongs to the archaeal groups previously detected in this fracture zone [34]. It is possible that CO_2_ alone or together with SO_4_ are not sufficient to activate the archaeal community present in the fracture zone at 180 m depth in Outokumpu. The same effect was shown previously when methane of methanol was used [7]. It should be noted, however, that without the addition of any substrates, no archaeal sequences were obtained and that even if the changes in the profile of the active archaeal community is only small, the addition of substrates was still needed for active transcription of the 16S rRNA genes to occur.

Fungi are present in the groundwater of Outokumpu at 180 m depth (Figure 4C). Previously, Nyyssönen et al [5] showed that approximately 0.6% of the sequence reads obtained from metagenomics shot gun sequencing of water samples from the Outokumpu drill hole water column originating from 600 m, 1500 m and 2300 m depth belonged to Eukaryotes. However, our study is the first to show that fungi are also present in groundwater and that the fungi are actively transcribing the rRNA operon, because the sequence reads we have obtained contain the ITS1 region flanked by the 5.8S and 18S rRNA gene regions. These unspliced transcripts would not be detected without on-going active transcription, because in order to produce functioning ribosomes, the ITS regions are removed from the long transcript and the rRNA subunits are freed. Fungal communities in deep groundwater has been reported from Olkiluoto situated on the western coast of Finland, from depths between 100 m and 800 m [35] and from the deep saline groundwater from below 2200 m in the Pyhäsalmi Mine [36], but in general the pelagic fungal communities are quite scarce. Nevertheless, the fungal community profiles in different locations differ, with Sordariomycetes being the most commonly detected clade in Olkiluoto, Sordariomycetes and Eurotiomycetes in Pyhäsalmi Mine deep groundwater and an unidentified group belonging to the Ascomycetes in Outokumpu 180 m groundwater. The scarcity of fungi in the water may be due to their preference to attach to solid surfaces. Thus, as shown by Rajala et al. [39], a considerably higher number of fungal 5.8S rRNA genes found on the surface of carbon steel immersed in Olkiluoto groundwater compared to the number detected in the groundwater itself.

Despite an apparent minority of bacteria rapidly initiating cellular respiration activity in response to CO_2_ in Outokumpu deep groundwater, bacteria able to utilize C from carbonate over a longer period of time were detected (Figure 6, Figure 7). NanoSIMS experiments demonstrated that isolated pure cultured *Pseudomonas* sp. and *Burkholderia* sp. strains incorporate carbon from inorganic carbon source (carbonate) in to cell structures. Burkholderia were generally present at very low abundances, and were detected only from the sorted cells and from the RNA fraction of the fracture zone water on sampling day and in the RNA fraction of the stored, untreated water. However, the *Pseudomonas* sp. 6.9 isolate represented well the majority of the gammaproteobacterial bacteria detected in the RNA fraction of the treated water samples. In the original fracture fluid sample, a fraction of bacterial cells was shown to slowly incorporate the carbon from the provided carbonate into their cell structures. A greater proportion of the microbial community appeared to be able to perform this fixation of inorganic carbon than was expected based on the portion that was rapidly activated by the added CO_2_ detected by FACS. This subpopulation, consisting at least of heterotrophic *Pseudomonas* and *Burkholderiales* bacteria, may fix inorganic carbon for the benefit of the whole microbial community. The importance of inorganic carbon fixation by heterotrophs in deep, dark oceanic environments has previously been shown by Yakimov et al. [40], who by use of ^14^C-protein-SIP showed that heterotrophic proteobacteria were the major inorganic carbon fixing group of the microbial community. They also isolated gammaproteobacterial pure cultures from this habitat and demonstrated that these bacteria assimilated inorganic carbon under *in situ* conditions [40].

## 5. Conclusion

In this study, microbial community from an isolated fracture zone at 180 m depth in the Outokumpu Deep Drill Hole was examined for the presence of microbial groups able to utilize CO_2_. One of the important ecological roles of these microorganisms is to replenish the carbon pool of the microbial community that would otherwise be lost through metabolic activities. Our results show that inorganic carbon fixation is performed by a specific part of the microbial community. These carbon-fixing bacteria belong to either gamma- or betaproteobacterial clades, which generally have very versatile metabolic capabilities. Methanogens using CO_2_ and H_2_ as substrates for methanogenesis were detected and shown to become activated by availability of CO_2_. In addition, fungi were detected from the different treatments, but the activation of different fungal clades by the added substrates appeared to be sporadic. Ability of carbon recapture by microbial groups is important because carbon would otherwise be lost in form of carbon dioxide during fermentation processes, cell respiration or anaerobic methane oxidation. Thus, our results indicate a putatively important role of carbon recapture by the small population of microorganisms performing carbon fixation or recapture in this environment.
